# Expression profile of E‐cadherin, estrogen receptors, and P53 in early‐onset gastric cancers

**DOI:** 10.1002/cam4.931

**Published:** 2016-10-25

**Authors:** Fan Zhou, Yuanyuan Xu, Jiong Shi, Xing Lan, Xiaoping Zou, Lei Wang, Qin Huang

**Affiliations:** ^1^ Department of GastroenterologyDrum Tower Hospital Affiliated to Nanjing University Medical SchoolNanjingJiangsuChina; ^2^ Department of PathologyDrum Tower Hospital Affiliated to Nanjing University Medical SchoolNanjingJiangsuChina; ^3^ Xuzhou Medical UniversityXuzhouJiangsuChina; ^4^ Department of PathologyVA Boston Healthcare System and Harvard Medical SchoolBostonMassachusetts

**Keywords:** Early‐onset gastric cancer (EOGC), E‐cadherin, estrogen receptors (ERs), P53

## Abstract

Early‐onset gastric cancer (EOGC) is predominant in females, diffuse histology, and hereditary pattern. Germline mutation of *CDH1* and *p53* has been reported previously and female dominance was speculated to be associated with estrogen and its receptors. Expression of E‐cadherin, estrogen receptor *α* (ER *α*), estrogen receptor *β* (ER *β*), and p53 in EOGC remains unclear, which was the focus of this study, to assess clinical significance of their expression in EOGC. The expression of E‐cadherin, ER *α*, ER *β*, and p53 in tumors and normal tissues from surgically resected EOGCs was assessed by immunohistochemistry (*n* = 139) and Western blot (*n* = 7) methods, respectively. The expression in tumor tissues was significantly higher for ER *α*, ER *β*, and p53, but lower for E‐cadherin, compared to uninvolved mucosa. Positive staining of ER *β* and p53 was more frequently observed in younger patients with advanced TNM stages. For E‐cadherin, significant correlation was observed between the immunopositivity and TNM stages IA+IB. P53‐negative patients had significantly better outcomes than p53‐positive patients. Significant association between expression of E‐cadherin and histologic types was found in familial, but not in sporadic, EOGC. In conclusion, our results demonstrated E‐cadherin may have a role in initiation of EOGC and positive ER *β* and p53 expression may partially explained early‐onset and tumor progression of EOGC.

## Introduction

The incidence of gastric cancer varies between regions of the world, with more cases in eastern Asia 1. Recently, the data of the National Cancer Institute's Surveillance Epidemiology and End Results (SEER) program showed a significant rising incidence of EOGC (early‐onset gastric cancer) in both female and male patients, but with a conspicuous female gender predominance 2, 3. EOGC is a subtype of gastric cancer in patients younger than 45 years old. Approximately 10–20% of young gastric cancer patients have a positive family history 4, some of whom present with inherited gastric cancer predisposition syndromes. Although the underlying genetic events are not always known, EOGC may show *CDH1* gene germline mutations 5, 6, 7, encoding an aberrant form of E‐cadherin, a cardinal feature of hereditary diffuse gastric cancer (HDGC), as recently reviewed by Carneiro et al. 7. However, *CDH1* may partially explain EOGC 8, and more studies 9, 10 would suggest *p53* as a candidate mutated gene in EOGC.

The *p53* gene is present at very low levels in normal cells and involved in many cellular functions, including the regulation of apoptosis, cell proliferation, angiogenesis, and cell cycle 11, 12. A mutation of the *p53* gene is frequently observed during the development of numerous human malignancies 13, 14. Overexpression of p53 has been shown in numerous human tumors, and high levels of p53 protein have been correlated with malignant progression in colorectal tumors and lung carcinoma in advanced stages. In addition, overexpression of p53 has been shown to be independently related to poor prognosis in breast carcinoma 15, 16. However, few studies have been conducted to assess p53 expression in EOGC 13.

In terms of gender differences in EOGC, most studies attributed the female predominance to possible roles of estrogen receptors in the pathogenesis of EOGC 17. Since Tokunaga et al. 18 first reported estrogen receptor (ER) *α* expression in gastric cancer, a series of studies have been focused on the role of ER*α* in gastric cancer progression. In 1996, two forms of ERs, ER*α* and ER*β*, were identified, but only ER*β*, not ER*α*, was expressed in gastric cancer 19, whereas others show the evidence of expression for both ER*α* and ER*β* receptor genes 19, 20, 21. Recently, a large Chinese cohort study 21 shows the presence of ER*α*, ER*β*, progesterone receptor (PR), and androgen receptor (AR) in both gastric cancer and noncancer tissues with predominant expression in ER*β* and no prognostic significance for the expression.

Herein in this study, we investigated the expression and clinicopathological significance of E‐cadherin, p53 in EOGC, and explored the role of ER*α* and ER*β* in EOGC progression in young Chinese patients treated at a single high‐volume hospital in China. To our knowledge, this study was the largest sample study regarding the predictive significance of E‐cadherin, p53, and estrogen receptors in EOGC.

## Materials and Methods

### Patients and tissue samples

EOGC patients younger than 40 years old at Nanjing Drum Tower Hospital, Jiangsu, China, from Jan 2004 to Dec 2014 were enrolled. Patients without enough tissue sample or necessary clinicopathological information, or loss to follow‐up were excluded from the study. The study cohort was part of our previous study 22. The paired formalin‐fixed paraffin‐embedded tissue blocks (tumor and nontumor in the same case) were retrieved and recut for immunohistochemistry. Proteins were extracted in frozen matched tumor and nontumor tissues from our biobank at this hospital. The study protocol was approved by the Medical Ethics Committee of the Nanjing Drum Tower Hospital. Informed consent was obtained from all individual participants included in this study.

### Immunohistochemistry

Immunohistochemical (IHC) analysis for E‐cadherin, ER*α*, ER*β*, and p53 expression was performed on formalin‐fixed, paraffin‐embedded sections of surgical specimens. Briefly, sections were deparaffinized in xylene and rehydrated in gradient ethanol solutions up to distilled water. Endogenous peroxidase activity was blocked by 0.3% H_2_O_2_ in methanol for 20 min. The slides were immersed in 10 mM citric buffer (pH 6.0) with heating for 15 min for antigen retrieval. Nonspecific binding sites were blocked with 10% normal goat serum for 10 min. Then, sections were incubated in a humidified chamber overnight with primary antibody (listed as in Table S1). Immunostaining was visualized with DAB and hematoxylin counterstain.

Two experienced pathologists (JS, QH) independently reviewed the expression of the four antibodies without the knowledge of patients' clinicopathological parameters. The scoring for E‐cadherin and ER*β* (expressed at a high level) was based on the area intensity score method (AIS) 23. Intensity scores from 0 to 3, respectively, represented absent, weak, moderate, and strong positive immunostaining. The area scores from 0 to 4 were estimated for the proportion of positively stained neoplastic cells in the entire tumor on the slide, as 0 = <5%, 1 = 5–24%, 2 = 25–49%, 3 = 50–74%, and 4 = ≥75%, respectively. The overall AIS score was obtained by multiplication. For ER*α* and p53 immunostaining, a negative stain was defined as less than 10% positive neoplastic cells on the slide; otherwise the stain was classified to be positive. Overexpression of p53 generally reflects an underlying mutation(s) in the *p53* gene, and manifests as positive immunostaining.

### Western blot analysis

Target tissues were homogenized in the RIPA lysis buffer. The supernatant was used for Western blot analysis. Protein concentrations were determined using the BCA assay regent. Thirty to sixty micrograms of protein lysates were separated on 6–12% sodium dodecyl sulfate‐polyacrylamide gels and then transferred to the PVDF membranes (Millipore). TBST (TBS and 0.1% Tween‐20) containing 5% nonfat milk or bovine serum albumin was used to block nonspecific binding for 2 h at room temperature. Then, the membranes were incubated with the primary antibodies against ER*α*, ER*β*, E‐cadherin, and p53 (detailed information is shown in Table S1). The membranes were rinsed three times with TBST for 10 min and reincubated for 1 h at room temperature in blocking buffer with each HRP‐conjugated secondary antibody (1:5000), and then washed three times for 10 min each. Signals generated by enhanced chemiluminescence (Millipore) were recorded with a CCD camera (CLINX, Shanghai).

### Statistical analysis

Difference in expression of E‐cadherin, estrogen receptors, and p53 between gastric tumors and corresponding uninvolved mucosal tissues was compared by the Students' *t* test or Wilcoxon matched‐pairs signed‐rank test where appropriate. Correlations were computed using the Spearman rank test. The associations between expression of E‐cadherin, estrogen receptors, and p53 and clinicopathological characteristics were analyzed using the Chi‐square test. The probability of survival was estimated by Kaplan–Meier method with a log‐rank test. All *P* values were two sided and considered statistically significant if less than 0.05. Statistical analyses were performed by the SPSS 22.0 for Windows (SPSS, Chicago, IL).

## Results

### Protein expression

**Figure 1:**
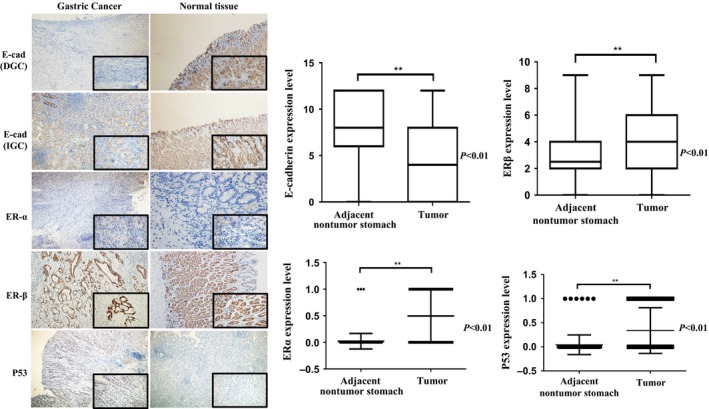
Immunostaining and analysis result of E‐cadherin, ER *α*, ER *β*, and P53 in Early‐Onset Gastric Cancers and Corresponding Adjacent Normal Tissues. DGC, diffuse gastric cancer; IGC, intestinal gastric cancer.

By immunohistochemistry performed in 139 EOGC tumors, expression of E‐cadherin was absent in 36 (25.9%), aberrant in 43 (30.9%), and normal in 60 (43.2%) patients, significantly lower than those (2.2%, 18.7%, and 79.1%, respectively) in uninvolved mucosal tissue (*P *<* *0.01) (Fig. 1, Table 1). In contrast, the expression of ER*α* (69, 49.6%), ER*β* (122, 72.2%), and p53 (42, 33.8%) in tumor tissues was significantly higher than those (2.2%, 60.3%, and 4.3% for ER*α*, ER*β*, and p53, respectively, detailed data for normal mucosal not shown) in uninvolved mucosal tissues (*P *<* *0.01). In terms of location, staining of E‐cadherin and p53 is consistent with previous studies, membranous and nuclei expression was demonstrated. Nuclei staining with anti‐ER*α* antibody was seen. While for ER*β*, EOGC was stained in both cytoplasmic and nuclei.

**Table 1:** Association between expression of E‐cadherin, ER*α*, ER*β*, and P53 and clinicopathological characteristics in early‐onset gastric cancer

Clinicopathologic Characteristics		Cases(*n* = 139)	E‐cadherin				ER*α*			ER*β*			P53		
			Absent	Aberrant	Normal	*P*	Positive	Negative	*P*	Positive	Negative	*P*	Positive	Negative	*P*
Gender	M	52	14 (38.9)	18 (41.9)	20 (33.3)	0.663	21 (30.4)	31 (44.3)	0.092	48 (39.3)	4 (23.5)	0.207	15 (31.9)	37 (40.2)	0.339
	F	87	22 (61.1)	25 (58.1)	40 (66.7)		48 (69.6)	39 (55.7)		74 (60.7)	13 (76.5)		32 (68.1)	55 (59.8)	
Age		33.8 ± 5.47	35.0 ± 4.01	32.5 ± 6.61	33.9 ± 5.22	0.126	33.9 ± 5.63	33.7 ± 5.36	0.858	33.6 ± 5.71	35.3 ± 3.06	**0.005**	32.2 ± 6.79	34.6 ± 4.48	**<0.001**
Family history		34	8 (24.2)	14 (32.6)	12 (20.7)	0.393	18 (26.9)	16 (23.9)	0.691	27 (23.1)	7 (41.2)	0.192	9 (20.5)	25 (27.8)	0.360
Size (cm)		4.6 ± 2.82	4.39 ± 3.31	4.84 ± 2.40	4.51 ± 2.82	0.751	4.79 ± 2.98	4.37 ± 2.67	0.387	4.69 ± 2.87	3.76 ± 2.42	0.509	4.95 ± 3.04	4.39 ± 2.70	0.805
*Hp* infection		62	17 (48.6)	20 (50.0)	25 (49.0)	0.992	28 (44.4)	34 (54.0)	0.285	54 (49.1)	8 (50)	0.946	19 (44.2)	43 (51.8)	0.417
Lauren's classification															
Diffuse		100	27 (84.4)	31 (79.5)	42 (72.4)	0.403	49 (74.2)	51 (81.0)	0.361	87 (76.3)	13 (86.7)	0.566	34 (81)	66 (75.9)	0.516
Intestinal		13	2 (6.3)	3 (7.7)	8 (13.8)	0.564	7 (10.6)	6 (9.5)	0.838	12 (10.5)	1 (6.7)	0.992	2 (4.8)	11 (12.6)	0.280
Mixed		16	3 (9.4)	5 (12.8)	8 (13.8)	0.888	10 (15.2)	6 (9.5)	0.332	15 (13.2)	1 (6.7)	0.764	6 (12.8)	10 (11.5)	0.652
LVI		80	16 (44.4)	33 (76.7)	31 (51.7)	**0.007**	42 (60.9)	38 (54.3)	0.432	73 (59.8)	7 (41.2)	0.145	37 (78.7)	43 (46.7)	**<0.001**
PNI		90	19 (52.8)	32 (74.4)	39 (65)	0.134	46 (66.7)	44 (62.9)	0.638	82 (67.2)	8 (47.1)	0.103	40 (85.1)	50 (54.3)	**<0.001**
LN		94	19 (52.8)	33 (76.7)	42 (70)	0.067	47 (68.1)	47 (67.1)	0.902	85 (69.7)	9 (52.9)	0.167	41 (87.2)	53 (57.6)	**<0.001**
Stage															
IA+IB		31	13 (36.1)	4 (9.3)	14 (23.3)	**0.017**	16 (23.2)	15 (21.4)	0.803	23 (18.9)	8 (47.1)	**0.009**	2 (4.3)	29 (31.5)	**<0.001**
IIA+IIB		31	7 (19.4)	13 (30.2)	11 (18.3)	0.321	15 (21.7)	16 (22.9)	0.874	29 (23.8)	2 (11.8)	0.422	9 (19.1)	22 (23.9)	0.523
III		65	12 (33.3)	22 (51.2)	31 (51.7)	0.172	32 (46.4)	33 (47.1)	0.928	60 (49.2)	5 (29.4)	0.126	30 (63.8)	35 (38)	**0.004**
IV		12	4 (11.1)	4 (9.3)	4 (6.7)	0.741	6 (8.7)	6 (8.6)	0.979	10 (8.2)	2 (11.8)	0.976	6 (12.8)	6 (6.5)	0.357

Bold values (*P *<* *0.05) are statistically significant.

Hp, Helicobacter pylori; LVI, lymphovascular invasion; PNI, perineural invasion; LN, positive lymph node metastasis.

**Figure 2:**
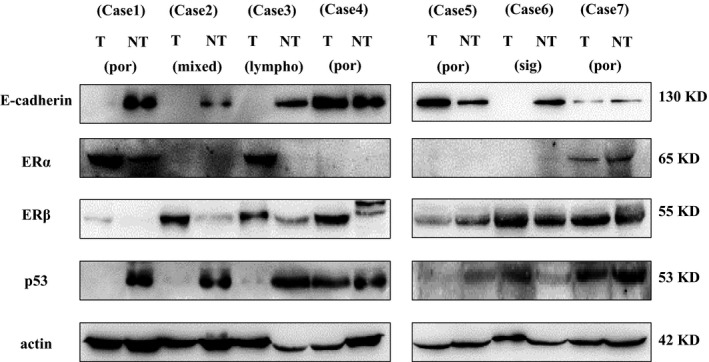
Western blot of E‐cadherin, ER *α*, ER *β*, and P53 in Seven Paired Early‐Onset Gastric Cancers and Corresponding Adjacent Normal Tissues. por, poorly cohesive carcinoma; lympho, lymphoepithelioma‐like gastric carcinoma due to EBV infection; sig, signet ring carcinoma.

Western blotting in seven paired EOGC and uninvolved mucosal tissues showed the patterns of changes similar to those by immunohistochemistry (Fig. 2). Due to *p53* mutation in gastric cancer, those mutated cases showed no signal in western blot.

### Correlation with clinicopathological characteristics

As shown in Table 1, for E‐cadherin, significant correlations were observed between the positive expression and TNM stages at IA+IB (*P *=* *0.017). And interestingly, absent E‐cadherin expression is significantly associated with lower lymphovascular invasion (LVI) (*P *=* *0.007). Importantly, protein expression of ER*β* and p53 was significantly associated with age and TNM stage, respectively. Positive staining of ER*β* and p53 was significantly more frequently observed in younger patients with advanced TNM stages (*P *<* *0.01). P53 expression is also significantly related to LVI, perineural invasion (PNI), and lymph node metastasis (*P *<* *0.001). No significant associations were found between expression of ER*α* and clinicopathological features.

### Prognostic value

**Figure 3:**
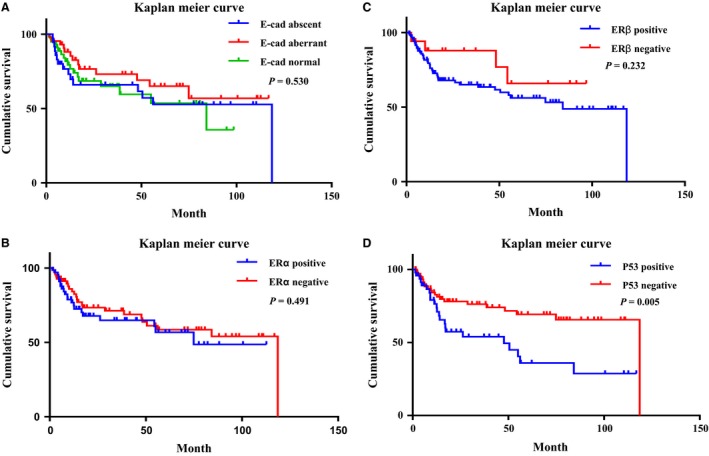
Kaplan–Meier Curve according to (A) E‐cadherin, (B) ER *α*, (C) ER *β*, and (D) P53 immunostaining (log‐rank test).

In all 139 patients with postresection survival information, expression of E‐cadherin, ER*α*, ER*β*, and p53 was analyzed for prognostic values by the Kaplan–Meier method. P53‐negative patients had a significantly better outcome than p53‐positive patients (*P *=* *0.005; Fig. 3D). However, expression of E‐cadherin, ER*α*, and ER*β* showed no prognostic values in EOGC patients (Fig. 3A, B, and C). Univariate Cox regression (Table 2) showed that higher CA 72‐4, CA 125, and CA 19‐9 level, larger tumor size, positive resection margin, LVI, PNI, advanced staging, and positive p53 expression were related with worse prognosis of EOGC. While for multivariate analysis, only CA 72–4 (RR: 4.622, 95% CI: 1.487–14.369, *P *=* *0.008), larger tumor size (RR: 1.139, 95% CI: 1.000–1.296, *P *=* *0.05), positive resection margin (RR: 5.718, 95% CI: 1.797–18.189, *P *=* *0.003), and stage IV (RR: 20.119, 95% CI: 1.486–272.465, *P *=* *0.024) are independent prognostic factors in EOGC.

**Table 2:** Univariate and Multivariate Analyses (Cox Regression) on Prognosis of Early‐onset gastric cancer Patients

Factors	Univariate analysis		Multivariate analysis	
	RR (95% CI)	*P*	RR(95% CI)	*P*
Female	1.340 (0.727–2.470)	0.348		
Age	1.001 (0.950–1.055)	0.713		
Positive Family history	0.769 (0.366–1.617)	0.489		
Higher CA 72–4	3.185 (1.473–6.886)	0.003	4.622 (1.487–14.369)	0.008
Higher CA 125	3.701 (1.486–9.216)	0.005		
Higher CA 19–9	4.241 (2.016–8.918)	<0.001		
Larger Tumor size (cm)	1.430 (1.295–1.580)	<0.001	1.139 (1.000–1.296)	0.05
Positive resection Margin	5.617 (2.831–11.143)	<0.001	5.718 (1.797–18.189)	0.003
Lymphovascular invasion	7.556 (2.960–19.288)	<0.001		
Perineural invasion	6.629 (2.583–17.013)	<0.001		
Staging I				
II	2.722 (0.283–26.221)	0.386		
III	22.488 (3.053–165.661)	0.002		
IV	69.400 (8.597–560.260)	<0.001	20.119 (1.486–272.465)	0.024
E‐cadherin expression	1.018 (0.701–1.480)	0.924		
ER*α* expression	1.230 (0.682–2.216)	0.492		
ER*β* expression	1.854 (0.663–5.185)	0.239		
P53 expression	2.269 (1.262–4.077)	0.006		

RR, relative risk; CI, confidence interval.

### Comparison in expression between tumor and uninvolved mucosal tissues

Table 3 shows the Spearman correlations in expression of E‐cadherin, ER*α*, ER*β*, and p53 between cancer and adjacent uninvolved tissues. Significant correlation was identified for expression of only E‐cadherin but not ER*α*, ER*β*, and p53. However, the correlation coefficients of E‐cadherin expression were so small (*r* = 0.261) that the correlation was extremely weak.

**Table 3:** Correlations among expression of E‐cadherin, ER*α*, ER*β*, and P53 in gastric cancer and adjacent nontumor tissue

Correlation	*r* a	*P* value^b^
E‐cad (T) vs. E‐cad (NT)	0.261	0.002
ER*α* (T) vs. ER*α* (NT)	0.051	0.554
ER*β* (T) vs. ER*β* (NT)	0.022	0.798
P53 (T) vs. P53 (NT)	−0.03	0.722
ER*α* (T) vs. ER*β* (T)	−0.046	0.591

E‐cad, E‐cadherin.

a Spearman rank correlation coefficients

b Spearman rank correlation test.

### Expression in diffuse and intestinal familiar and sporadic gastric cancers

As shown in Table 4, expression of E‐cadherin was significantly more frequent in intestinal and diffuse mixed EOGC tumors of familiar, (*P *<* *0.05), but not sporadic EOGC cases. No significant associations were found between expression of ER*α*, ER*β*, and p53 and different histology types of EOGC.

**Table 4:** Expression of E‐cadherin, ER*α*, ER*β*, and P53 in diffuse and intestinal FGC and SGC

Clinicopathologic Characteristics	E‐cadherin				ER*α*			ER*β*			P53		
	Absent	Aberrant	Normal	*P* value	Positive	Negative	*P* value	Positive	Negative	*P* value	Positive	Negative	*P* value
**FGC**				**0.03**			1.000			1.000			0.288
Diffuse+Mixed	7 (87.5)	13 (100)	7 (63.6)		15 (83.3)	12 (85.7)		22 (84.6)	5 (83.3)		9 (100)	18 (78.3)	
Intestinal	1 (12.5)	0	4 (36.4)		3 (16.7)	2 (14.3)		4 (15.4)	1 (16.7)		0	5 (21.7)	
**SGC**				0.695			1.000			1.000			0.932
Diffuse+Mixed	20 (95.2)	23 (88.5)	41 (91.1)		42 (91.3)	42 (91.3)		75 (90.4)	9 (100)		28 (93.3)	56 (90.3)	
Intestinal	1 (4.8)	3 (11.5)	4 (8.9)		4 (8.7)	4 (8.7)		8 (9.6)	0		2 (5.7)	6 (9.7)	

Bold values (*P *<* *0.05) are statistically significant.

FGC, familial gastric cancer; SGC, sporadic gastric cancer.

## Discussion

Our study reveals that expression of E‐cadherin, estrogen receptors, and p53 is significantly altered in EOGC. As expected, expression of E‐cadherin was decreased in the diffuse‐type familial EOGCs, whereas positive ER*β* and p53 expression correlated with younger age and advanced TNM stages in EOGC, which has not been described previously. Independent prognostic factors in EOGC were higher CA 72–4 level, larger tumor size, positive resection margin, and stage IV cancer.

Although decreased expression of E‐cadherin is known in the diffuse‐type gastric cancer in general, aberrant expression of this gene is widely present in EOGC regardless of histological type 24, but also correlated significantly with the diffuse and mixed types in the familial gastric cancer (FGC) 25, suggesting a role of E‐cadherin in FGC tumorigenesis. We showed that E‐cadherin expression was not associated with gender, age, family history of cancer, tumor location, size, and *Hp* infection, except for stages IA+IB. This suggests that E‐cadherin may play a critical part in the early‐stage tumorigenesis of EOGC 26. However, we also reported that absent E‐cadherin expression was associated with lower LVI rate compared to aberrant and normal group. This phenomenon may be explained by mesenchymal to epithelium transition (MET)^27^. E‐cadherin is important in cellular junction and maintenance of epithelial phenotype. When E‐cadherin switched to N‐cadherin, epithelial–mesenchymal transition (EMT) happens. After tumor cells spread to targeted region, disseminated tumor cells undergo MET. E‐cadherin is regulated by both transcriptional and epigenetic mechanism. As our study was limited to the protein level of E‐cadherin expression, further analysis at gene levels and epigenetic level may help reveal the molecular pathogenesis mechanisms for E‐cadherin in a large sample Chinese EOGC cases 28.

*p53* is one of the most important genes that are mutated in gastric cancer 29, 30. In accordance with the previous reports 31, patients with p53‐positive and HIF‐1*α*‐positive gastric cancer have worse prognosis, compared with those with double negative cancers. Our study on EOGC further suggests that p53‐negative patients have a significantly better outcome than p53‐positive patients, which is in disagreement with a most recent Turkish report because of the different study population and a high percentage of elderly patients investigated 32. We show that overexpression of p53 is associated with younger age but advanced stage of EOGC, suggestive of an aggressive biology behavior. And positive expression was associated with worse prognosis. However, multivariate analysis suggested it was not an independent factor. A recently one study 33 from Korean reported that overexpression of p53 is less frequent in younger GC patients. The inconsistency may be explained by the selection of patients. We did not include older GC patients who are reported to have a high *p53* mutation rate 9.

Relative to old gastric cancer patients, young patients have a female preponderance, a more frequent occurrence of the diffuse‐type cancer, and less intestinal metaplasia 4, 24, 34. This female gender predominance in EOGC is considered to be related to hormonal factors 35, 36. While our results show an absence of a significant correlation between ER*α* expression and clinicopathologic parameters, ER*β* expression is indeed correlated with younger age and advanced cancer stages. In conventional gastric cancer with a high proportion of elderly patients, expression of ER*α* correlates with poor overall survival, as an independent predictor of overall survival 20, which is not confirmed in EOGC, suggesting different pathogenesis mechanisms between conventional gastric cancer and EOGC. Matsuyama et al. 19 reported that among signet ring carcinoma, ER*β* cytoplasm was stained in addition to nuclei, especially in EOGC, which is exactly what we observed in this study. The exact role of cytoplasmic ER*β* remains unclear, but because low expression level of ER*α*, we can conclude that ER*β* may mediate the estrogen effect in stomach 37.

A major limitation of our study is that we focused on only protein alterations of E‐cadherin, ER*α*, ER*β*, and p53 in EOGC. Therefore, the results remain to be validated by further investigations of genetic mutations of EOGC at DNA and RNA levels. Many EOGC patients diagnosed at advanced stages with extensive metastasis were excluded because of palliative management without surgery. The current results may be biased, which remain to be corrected in the upcoming investigation of familiar gastric cancer.

In conclusion, our study demonstrated significant differences in expression of E‐cadherin, estrogen receptors, and p53 between EOGC tumors and adjacent uninvolved mucosal tissues. Expression of E‐cadherin was significantly associated with the diffuse‐type familial EOGCs and early stage, whereas expression of ER*α* might have little role in EOGC tumorigenesis. Expression of ER*β* and p53 significantly correlated with age and advanced cancer stages, and the p53‐negative EOGC was associated with favorable outcomes. Further studies are needed to explore the different molecule genetic profile of EOGC from that of conventional gastric cancer occurring at a later age.

## Conflict of Interest

None.

## Supporting information


**Table S1.** Antibodies used in this study.
