# Supplementary material for: Expression profile of E‐cadherin, estrogen receptors, and P53 in early‐onset gastric cancers
Source: Cancer Med. 2016 Oct 25;5(12):3403–11. doi: 10.1002/cam4.931 (PMC5224840; doi:10.1002/cam4.931)
Supplement: Supplementary file 1 — Table S1. Antibodies used in this study. [file CAM4-5-3403-s001.docx]

Supplementary Table 1. Antibodies used in this study

|  |  | Immunohistochemistry | |  | Western Blot | |
| --- | --- | --- | --- | --- | --- | --- |
|  | Manufacture | Antibody | Concentration | Manufacture | Antibody | Concentration |
| E-cadherin | Abcam | HECD-1 | 1:200 | Abcam | HECD-1 | 1:1000 |
| ERα | Abcam | SP1 | 1:400 | Abcam | SP1 | 1:1000 |
| ERβ | Santa Cruz | H-150 | 1:200 | Abcam | 14C8 | 1:1000 |
| p53 | CST | 7F5 | 1:1000 | CST | 7F5 | 1:1000 |
| β-actin |  |  |  | Sigma | AC-15 | 1:5000 |

CST, cell signal technology
